# Assessing and Addressing the Determinants of Appalachian Population Health: A Scoping Review

**DOI:** 10.13023/jah.0503.07

**Published:** 2023-12-01

**Authors:** David L. Driscoll, Hannah O’Donnell, Maitri Patel, David C. Cattell-Gordon

**Affiliations:** Healthy Appalachia Institute, UVA Wise; UVA Health; UVA School of Medicine; Center for Telehealth at the University of Virginia (retired); ThreadEx Consulting LLC (current)

**Keywords:** Appalachia, SDOH, social determinants of health, scoping review, Virginia

## Abstract

**Introduction:**

Residents of Appalachia experience elevated rates of morbidity and mortality compared to national averages, and these disparities are associated with inequitable exposures to various determinants of population health. Social and environmental determinants of health are a useful lens through which to develop and evaluate programs to mitigate regional health disparities.

**Methods:**

This 2023 scoping review was conducted of studies linking determinants of Appalachian health with leading causes of regional mortality and morbidity. The search strategy employed a keyword search that included geographic terms for the Appalachian Region and the primary adverse health outcomes in that region. Studies meeting the following inclusion criteria were reviewed: original article, published in the last five years, involving an Appalachian population, and includes a rigorous assessment of an association between a population health determinant and one or more leading causes of Appalachian morbidity and mortality.

**Results:**

The search returned 221 research articles, including 30 interventional studies. The top three health outcomes included cancer (43.59%), diseases of despair (23.08%), and diabetes (12.82). Access to care (27.3%), rurality (18.9%), and education (14.8%) were the most common population health determinants identified. Interventional studies were categorized by program types: education, technology, partnerships, and multilevel interventions. Due to the heterogeneity of study types, the studies were combined using a narrative synthesis.

**Implications:**

The results of this work can inform the development and evaluation of additional programs to promote Appalachian population health. Our study team will use these results to inform community-based discussions that develop strategic plans to mitigate health disparities in Central and Southcentral Appalachian Virginia.

## INTRODUCTION

The roughly 25 million residents of the 13-state Appalachian Region^1^ experience significantly higher rates of preventable health problems, including heart disease, cancer, stroke, and diabetes, compared to state and national averages.^2^ Appalachian residents also experience greater inequities related to the underlying causes of illness—the social and environmental determinants of health—including educational attainment, employment, income, and access to health care.^2^–^4^ The determinants of health are a useful lens through which to develop and evaluate programs to mitigate regional health disparities.^5^ The COVID-19 pandemic has put a tremendous strain on rural healthcare systems, the economy, and the residents of Appalachia. There is a need for investigation of the immediate pre- and post-pandemic determinants of population health in Appalachia, as well to assess recent evidence-based interventions addressing them.

### Study Objectives

Faculty and staff associated with the Healthy Appalachia Institute (HAI) and the Center for Telehealth at the University of Virginia conducted a scoping review to assess what determinants of Appalachian population health were described in the recent published literature. This scoping review is part of an ongoing project to inform the research portfolio at the HAI and contribute to the development of a regional Blueprint for Health Improvement and Health-Enabled Prosperity for the Central and Southcentral Appalachian regions of Virginia. These regions include the westernmost 13 counties in Virginia, of which four are defined as economically distressed and six as economically at-risk by the ARC.^3^ The project had two overarching objectives:

1. Identify studies that demonstrate associations, either positive or negative, between determinants of population health and leading Appalachian health outcomes, and
2. Characterize evidence-based programs or policies that have been demonstrated to modify one or more determinants of Appalachian population health.

## METHODS

A scoping review of Appalachian health literature was conducted to assess the latest evidence on population health determinants in Appalachia. Scoping reviews are conducted over systematic reviews when the main purpose of the review is to identify gaps in existing knowledge. The methods include identifying and clearly defining the research question, searching for and identifying relevant studies, selecting studies, charting the data both quantitatively and thematically, and summarizing the results.^6^,^7^ The specific scoping review goals were to examine the Appalachian health literature thematically, to synthesize recent assessments of population health determinants in the region, and to identify research that points to exemplary programs for potential implementation in Central and Southcentral Appalachian regions of Virginia.

### Leading Adverse Health Outcomes

The leading causes of mortality (1–10) in the Lenowisco, Cumberland Plateau, and Mount Rogers health districts of Virginia were identified using the CDC WONDER online database^8^. Oral Health (11) was added to the search parameters as an important comorbidity in the region:

1. Heart disease
2. Malignant neoplasms
3. COVID-19
4. Chronic respiratory disease (including black lung)
5. Unintentional injuries
6. Diabetes mellitus
7. Cerebrovascular diseases
8. Alzheimer’s disease
9. Influenza and pneumonia
10. Diseases of Despair:
  - Chronic liver disease and cirrhosis
  - Intentional self-harm (suicide)
  - Poisoning/drug overdose
11. Oral Health

### Population Health Determinants

Draft determinants of Appalachian health were identified for the initial coding process using data from the University of Wisconsin Population Health Institute’s County Health Rankings,^9^ the 2008 ARC Report,^10^ and archival data from the 2011 and 2016 Southwest Virginia Blueprints for Health Improvement and Health-Enabled Prosperity.^11^,^12^ This list was augmented and expanded during the scoping review process.

### Search Strategy

A comprehensive search strategy was developed through a collaborative and iterative process between the researchers. The search was performed using the Web of Science (WoS). This citation indexing service provides access to content in the life, biomedical, and social sciences field, such as books, journals, conference proceedings, workshops, and professional reports. The wide scope and coverage of this database likely includes most of population health research conducted in Appalachia. The search included keywords related to Appalachia and the leading causes of mortality in the Central and Southcentral Appalachian regions of Virginia. Additional publications recommended by key stakeholders, specifically local health care and social services providers in the region, were added if they met the search criteria. There were no restrictions placed on language. All searches were conducted between February 1, 2023, and April 30, 2023.

### Inclusion Criteria

The following inclusion criteria were used: (1) original article written by the person or people who conducted the research; (2) published in the last five years (2018 to 2023); (3) involving an Appalachian population; and (4) includes a rigorous assessment of an association between a social or environmental determinant and one or more leading causes of Appalachian morbidity and mortality.

### Data extraction

Three authors (DD, HO, MP) undertook the database search, exported all citations into Zotero, and conducted the initial screening of the titles and abstracts. Where uncertainty existed in relation to potential eligibility, titles and abstracts were reviewed as a group, and ambiguities or disagreements were resolved through discussion.

All the authors divided the health outcomes into four groups for the full-text review and began identifying the population health determinants. Uncertainties were resolved during full team discussions, and the authors reached a consensus. The following information from each included study was extracted: related health outcome, type of study, involvement of a pediatric population, testing of intervention, effectiveness of intervention, a brief description of the intervention, corresponding author institution, state(s) where the study was conducted, and applicable population health determinants. The interventional studies were then categorized by program type.

## RESULTS

### Search Results

**Figure 1:**
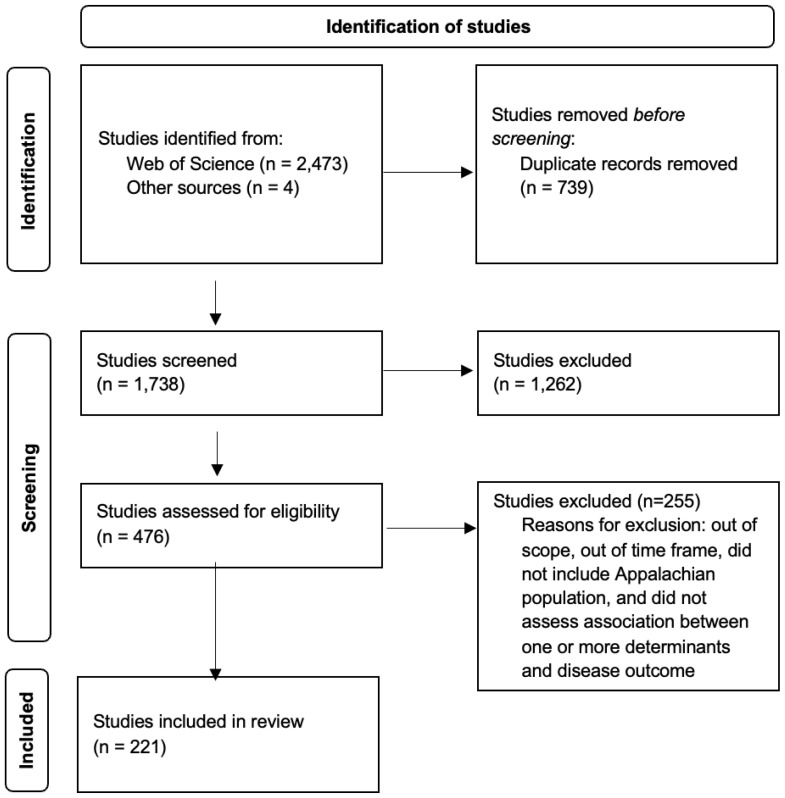
PRISMA-P flow diagram NOTE: This diagram was adapted from Page MJ, McKenzie JE, Bossuyt PM, Boutron I, Hoffmann TC, Mulrow CD, et al. The PRISMA 2020 statement: An updated guideline for reporting systematic reviews. *BMJ* 2021;372:n71.^11^

The WoS database search and articles identified through other sources returned 2,477 titles and abstracts, of which 476 potentially relevant studies were subjected to full-text review, and 221 (full list available upon request) were ultimately found to be eligible for inclusion. A PRISMA flow chart summarizes the search strategy (Fig. 1).

### Study Characteristics

The top three study types were cross-sectional (36.65%), mixed methods (22.17%), and cohort (20.36%). There were 23 (10.41%) qualitative studies, not including mixed-method designs, and 18 (8.14%) case-control studies. There were two randomized control trials (RCT). Over 80% of the included studies were analytic. The studies primarily (90.05%) focused on adult populations.

The studies were made up of the following health outcomes: cancer (43.59%), diseases of despair (23.08%), diabetes (12.82), respiratory disease (7.69%), COVID-19 (5.13%), diet/nutrition/exercise (2.56%), oral health (2.56%), and stroke (2.56%). The search did not return any studies related to influenza or pneumonia.

### Corresponding Author Institution

**Figure 2:**
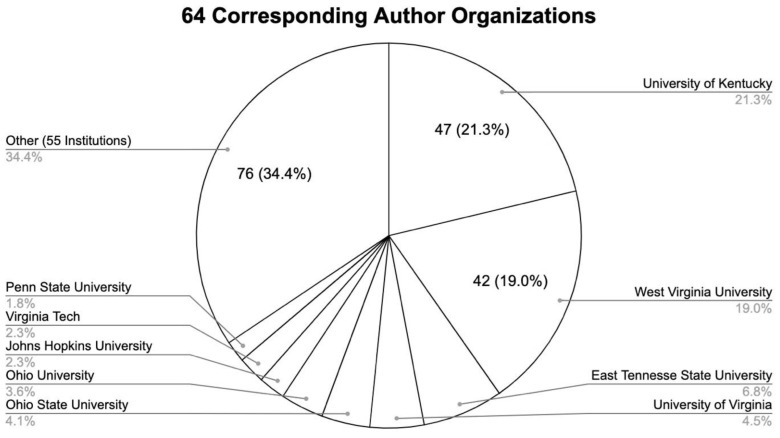
Breakdown of corresponding author organizations

The studies included 64 corresponding author institutions. The University of Kentucky (21.3%), West Virginia University (19%), and East Tennessee State University (6.8%) made up the majority of the studies. Fig. 2 provides a full breakdown of the corresponding author organizations.

### Population Health Determinants

One of the objectives of this scoping review was to synthesize recent assessments of population health determinants in the region. These included *Access to care*, or adequate and timely access to comprehensive personal health services; *rurality*, or geographic, topographical, and social, economic, and political barriers to services; and *education*, or access to quality instruction and training, as the most frequently assessed determinants of Appalachian health (at 27%, 19%, and 15%, respectively). These were followed by employment/income/poverty (12.7%); substance use disorder (12.1%); diet/exercise/nutrition (8%); occupational conditions (2.7%); environmental conditions (2.5%); and traumatic stress (1.1%). Definitions of these nine determinants can be found in the Additional Files section. Table 1 shows the frequency of each determinant per health outcome.

### Interventional Studies

The scoping review included 30 successful interventions among the final list of studies for full review, or roughly 14% of the total. These interventions included *educational programs*, which demonstrated successful techniques for promoting informed health decision-making among Appalachian residents; *technological applications*, which demonstrated the effectiveness of a variety of telehealth programs, machine learning solutions, and innovative screening or testing methods; and *collaborative partnerships*, which described successful combinations of investigators, health service providers, and community residents and stakeholders. Finally, several *multilevel interventions* were identified that included some combination of all three categories of programs, such as educational programs using technological applications.

#### Educational programs

The scoping review included 11 evidence-based educational programs.^12^–^23^ As one may imagine, all these interventions promoted access to health-focused instruction and training. In addition, several of these programs increased access to care by promoting opportunities for vaccination, or health-related screening, often for cancer. For example, Paskett et al. demonstrated how motivational interviewing among Appalachian patients can promote human papillomavirus vaccinations and testing as a way to reduce cervical cancer disparities in Appalachia.^14^ Kelly et al. demonstrated how a pharmacy-based dynamic communication model can decrease unprotected exposure to sunlight and promote screening for skin cancer among residents of rural West Virginia.^20^ Other educational programs focused on promoting healthy diet and nutrition and reducing substance use. For example, Ickes et al. engaged youth advocates to promote tobacco control efforts in their communities, and in so doing, reduce the disparity in youth smoking in Appalachian Kentucky,^21^ while Kids SIPsmartER, a school-based intervention, effectively reduced the use of sugar-sweetened beverages in children and caregivers in the Appalachian region of Virginia.^22^

#### Technology

Ten studies utilized technological applications to population health challenges, such as telehealth services, machine learning, and automated electronic medical record (EMR) reminders to improve access to healthcare in rural communities.^23^– ^33^ Geographic, financial, and cultural barriers to access to health care— including screening, diagnosis, monitoring, and disease management—were particularly relevant to the diseases of despair and cancer. For example, Saeed, Jones, and Muppavarapu demonstrated that offering telepsychiatry services in emergency departments promotes access to care, improves outcomes, and reduces unnecessary hospitalizations among substance use disorder patients.^23^ Ward et al. utilized machine-learning natural-language coding to reduce the time it takes to code overdose death certificates in Kentucky, which improved public health response rates.^24^

During the COVID-19 pandemic, the use of telemedicine increased substantially. Telemedicine was a useful tool to improve access to primary care for residents in rural areas and increased visit completion rates by 20%, out of a sample of 110,99 patient visits, including 13,013 telemedicine visits.^25^ The fast implementation of an outpatient tele-neurology program successfully expanded patient access to care during the COVID-19 pandemic.^26^ The use of an inexpensive online COVID-19 symptom checker in North Carolina enabled the collection of local COVID-19 surveillance data in the spring of 2020. This provided important situational information to local public health officials by highlighting shifts in COVID-19 symptom patterns, identifying the geographical spread of these changes, and pinpointing obstacles related to accessing health care and testing facilities.^27^

Zoellner et al. conducted a quality improvement project for CRC screening in federally qualified health centers (FQHCs) in Appalachian Virginia and found that there were no reported differences between completion rates of fecal immunochemical test (FIT) based on follow-up communication type. One group was delivered live telephone reminders by care coordinators (usual care) while the second group received EMR automated reminders. The use of automated patient reminders provides facilities with limited resources a helpful tool for maintaining standards of care.^28^

#### Partnerships

Four studies employed innovative community partners to promote access to health care in rural Appalachian communities. For example, Mitchell et al. employed lay navigators (LNs) to identify under-screened women in their community, provide information about cervical cancer screening recommendations, and distribute self-collection kits. 33 Crespo et al. used a similar model with community health workers (CHWs) to overcome limitations to health care by providing regular home visits to patients with chronic health problems, and in so doing, improved management of those diseases.^34^

Ostrach et al. and Winstanley et al. used an innovative community-led approach to substance use disorder by increasing access to buprenorphine-containing medications. In the former intervention, local county health departments in western North Carolina established exclusive dispensing agreements with independent community pharmacies. This arrangement guaranteed a consistent and reliable medication supply for patients who were prescribed buprenorphine, while decreasing participants’ experiences of stigma when interacting with pharmacies.^35^ In the latter intervention, West Virginia adopted a hub-and-spoke model to strengthen the organizational capabilities of healthcare facilities in utilizing buprenorphine for treating patients with OUD. This program enabled 14 healthcare facilities to provide MAT, 56 health professionals to be trained, and 196 additional patients to be treated.^36^

#### Multilevel interventions

Interventions that combined educational programs, technological applications, and/or innovative partnerships, seemed to provide particularly effective and sustainable population health solutions. Five studies used a multilevel approach.^37^–^41^ For example, Key et al. developed a Facebook-based educational intervention to promote healthy nutrition and exercise behaviors and access to health care by overcoming economic barriers to screening, and reduced modifiable colorectal cancer risk factors.^37^ Wallace and Behringer used iPad tablets and an online curriculum to help pastors in Tennessee to access consumer health databases and improve access to cancer care and management in their congregations. This combination of education, technology, and community partnerships to improve and educate local clergymen on cancer topics offers a sustainable method to reach community members who often look to their religious leaders for support.^38^

## DISCUSSION

This scoping review was intended to identify recent studies of associations between leading Appalachian health outcomes and their determinants, and to characterize those interventions that successfully modify one or more of the determinants. These findings demonstrate that many Appalachian health disparities are due to inequitable exposure to a variety of social and environmental determinants of population health, and that these determinants can be modified by evidence-based interventions. Further, these findings provide some insights into what data exist, as well as what data do not yet exist, for the development of future strategic population health improvement plans to reduce Appalachian health disparities.

The analysis suggests that current interventions that successfully modify Appalachian determinants fall into one or more of three broad programmatic categories: (1) those that provide educational materials to promote informed health decisions on the part of patients and/or their providers; (2) those that employ innovative technological applications; and (3) those that enhance collaborations with strategic community stakeholders and partners. It is noteworthy that many of the innovative technological applications described in the recent literature, particularly the expansion of telemedicine, were implemented in response to the COVID-19 pandemic. These innovations allowed for improved surveillance of COVID-19 symptoms and promoted improved access to health care for Appalachian residents. It is hoped that these innovative applications will continue, that additional categories can be added to this list of evidence-based strategies in the future, and that the programs described here can inform community-based efforts to refine causes, needs, and priorities in Appalachian communities. Such efforts will be crucial to the development and evaluation of effective and sustainable interventions in the region.

### Limitations

As with all scoping reviews, this study presents data contained in the scientific literature. We cannot suggest that these contributions capture the full array of Appalachian population health challenges, determinants, or potential interventions across the region. Further, the studies and programs described herein come from sources available through the WoS platform, which may not include every important contribution to the Appalachian health literature. In response to this limitation, the team presented these results to—and invited additional items from—colleagues and stakeholders in the study region, and in so doing, identified four additional studies for inclusion.

## IMPLICATIONS

The methodology used in this review provides a template for investigators seeking to address population health disparities in their local communities. The results of this review can be used to gather community-based data on local health priorities, and to develop and evaluate locally supported interventions. These community-based strategies can go some way to mitigating the first study limitation identified above by identifying previously unrecognized health problems, determinants, or interventions.

Our team is currently describing the results of this scoping review to residents of Central and Southcentral Appalachian Virginia to identify priority health challenges and solutions. We believe that doing so will promote greater local engagement and partnerships, as well as long-term sustainability of subsequent interventions. The results of this process will be documented in a 2023 iteration of the Southwest Virginia Blueprint for Health Improvement and Health-Enabled Prosperity, as well as shape the future research portfolio of the HAI at UVA Wise. We hope that the updated health Blueprint will inform state and local policy, including the allocation of resources to create or support programs addressing the social and environmental determinants of population health in Central Appalachia.

**SUMMARY BOX**

**What is already known about this topic?**

Residents of Appalachia face elevated rates of morbidity and mortality compared to the national averages, and these disparities are associated with inequitable exposures to various social and environmental determinants of population health leading to preventable adverse health outcomes.

**What is added by this report?**

This scoping review outlines the top population health determinants associated with leading Appalachian health outcomes. Additionally, it highlights evidence-based initiatives that have successfully addressed one or more determinants of Appalachian population health.

**What are the implications for future research?**

The findings will guide the development of the new Blueprint for Enhancing Health and Fostering Prosperity in Southwest Virginia. This scoping review also provides a template for other investigators developing strategic population health promotion plans in their regions.

## Supplementary Information



## Figures and Tables

**Table 1:** Numbers of studies associating determinants to adverse health outcomes

Outcomes	Heart Disease	Cancer	COVID-19	Respiratory Disease	Unintentional Injury	Diabetes	Stroke	Alzheimer’s Disease	Diseases of Despair	Oral Health	Total # of Studies*
Access to care	11	54	14	7	1	14	2	5	38	7	153
Rurality	7	42	7	6	1	10	2	3	25	3	106
Education	6	30	6	3	2	15	1	2	14	4	83
Substance Use Disorder	4	13	2	5	0	2	2	0	40	0	68
Income/Poverty	5	29	3	3	0	6	0	1	19	5	71
Diet/Nutrition/Exercise	12	8	3	2	0	13	1	0	2	2	43
Occupation	0	0	2	10	1	0	0	0	2	0	15
Environmental	1	5	1	4	0	1	0	1	1	0	14
Trauma	0	0	0	0	0	1	0	0	5	0	6

NOTES:

* Some studies assessed multiple determinants, and thus the sum exceeds 221.
