# Supplementary material for: Assessing and Addressing the Determinants of Appalachian Population Health: A Scoping Review
Source: J Appalach Health. 2023 Dec 1;5(3):85–102. doi: 10.13023/jah.0503.07 (PMC11110904; doi:10.13023/jah.0503.07)
Supplement: Supplementary file 1 [file 5.3.7_Driscoll_Additionalfile.docx]

**Supplementary Material**

**Population Health Determinant Definitions**

1. Access to care: Adequate and timely access to comprehensive personal health services, which include preventive, dental, and primary, secondary, and tertiary health care, to achieve the best possible health outcomes.
2. Rurality: Geographic distance, topographical barriers, and social, economic and political factors that result in persistent isolation and disenfranchisement from social support services and programs.
3. Education: Instruction and training of sufficient quality to achieve safe, high-paying jobs and allow informed health decision-making.
4. Substance Use Disorder: A problematic pattern of use of substances, including alcohol, tobacco products, drugs, inhalants, and other substances which lead to impairments in health, social function, and control over the substance or substances used.
5. Employment/Income/Poverty: Access to employment opportunities that provide financial resources sufficient to meet basic needs, a sense of purpose, and hope for advancement.
6. Diet/Nutrition/Exercise: Consumption of a nutritious diet and participation in physical activities sufficient to prevent obesity, maintain a healthy body mass index, and achieve physical fitness.
7. Occupational conditions: Hazards, demands, prestige, social networks, and other risks and benefits experienced in the occupational environment.
8. Environmental conditions: The quality, accessibility, and safety of the local environment including water, air, and climate.
9. Trauma: Traumatic experiences including abuse, neglect, and domestic violence either as adverse childhood experiences (ACEs) or in adulthood. In some cases, these are multi-generational experiences.
